# Barriers to vulnerable adolescent girls’ access to sexual and reproductive health

**DOI:** 10.1186/s12889-022-14687-4

**Published:** 2022-11-29

**Authors:** Mojgan Janighorban, Zahra Boroumandfar, Razieh Pourkazemi, Firoozeh Mostafavi

**Affiliations:** 1 Department of Midwifery and reproductive health, Nursing and Midwifery Care Research Center, Faculty of Nursing and Midwifery, Isfahan University of Medical Sciences, Isfahan, Iran; 2 Reproductive Sciences and Sexual Health Research Center, Isfahan University of Medical Sciences, Isfahan, Iran; 3 Nursing and Midwifery Care Research Center, Faculty of Nursing and Midwifery, Isfahan University of Medical Sciences, Isfahan, Iran; 4 Department of Health Education and Promotion, School of Health, Isfahan University of Medical Sciences, Isfahan, Iran

**Keywords:** Adolescent health, Sexual and reproductive health, Sexual and reproductive health services, Qualitative study

## Abstract

**Background:**

Vulnerable adolescents are exposed to sexual and reproductive health harms. Ignoring the sexual and reproductive health of this group can have irreparable consequences. The present qualitative study aimed to explore the barriers to the access of vulnerable adolescent girls to sexual and reproductive health.

**Methods:**

In this study, sixteen 14-19-year-old adolescent girls and twenty-two key informants were selected using purposive sampling method. Through in-depth semi-structured interviews, they expressed their experiences of barriers to sexual and reproductive health in vulnerable adolescent girls. The data were encoded using the conventional qualitative content analysis.

**Results:**

Based on the results of the study, neglecting the reproductive and sexual health of vulnerable adolescent girls at different levels leads to serious challenges and obstacles in providing and maintaining it. Lack of a responsible family, the repulsive behaviors of the family and following risky behaviors of peers led to ignoring the sexual and reproductive health of adolescent girls. Unanswered sexual questions, defective life skills, unwanted pregnancy during adolescence, lack of awareness of unsafe sex, violating cultural norms and wounded psyche in vulnerable adolescent girls threaten their sexual and reproductive health. Ineffectiveness of key organizations in providing sexual and reproductive health services alongside lack of legal, political and social support in this area indicate that the sexual and reproductive health of these girls is not a priority for the society.

**Conclusion:**

Numerous personal, family, social, legal and political barriers challenge the sexual and reproductive health of vulnerable adolescent girls. Developing a comprehensive and practical program beside legal and political support for this issue can provide the basis for the sexual and reproductive health of this group of adolescents in societies.

## Background

Adolescence is a time of personal experience and choice, when personal and sexual identities are formed. Becoming a sexually healthy adult is one of the key developmental activities in adolescents. Sexual health requires a positive and respectful approach to sexuality and sexual relationships, as well as a pleasurable and safe sexual experience, free of coercion, discrimination and violence [1].

Risk-taking and emotion-seeking together with the misconception of invulnerability in adolescence period can lead to increased assertiveness in engaging with risky behaviors [2]. High-risk behaviors are defined as behaviors with an adverse effect on the overall growth and health of adolescents that may prevent them from future progress and success. High-risk behaviors may include violent behaviors such as physical harm or behaviors such as alcoholism, smoking, high-risk sexual behavior, and use of narcotics [3].

In a study conducted on 385 14–19-years-old adolescents, 120 subjects (23.3% of them were female and 40.4% were male), were involved at least once in their lives, in sexual relations either voluntarily or by force. 19.5% of these adolescents were exposed to high-risk sexual behaviors and sexually transmitted diseases such as HIV [4].

Vulnerable adolescents are a special group who given to their personal, family, economic, social and cultural conditions are exposed to physical and mental activities which may threaten their sexual and reproductive health. Vulnerable adolescents in this study refer to the adolescent girls who use drugs (stimulants, alcohol and hallucinogens), have high-risk sexual behaviors, a history of sexual harassment, a history of running away from home, and those living in welfare centers, social emergency centers and drug hangouts. The adolescent girls involved in any of these behaviors or a combination of them are considered to be vulnerable. In the study of Garmaroudi et al. (2010), conducted in the Welfare Organization of Iran, 50% of street women referred to rehabilitation centers were 15–19 years old and 24% of them were in the age range of 19–24 years [5].

Adolescents are among the most important target groups in sexual and reproductive health programs. Sexual relations, especially unprotected ones, are associated with irreparable consequences, such as infection with HIV and other sexually transmitted diseases, unwanted pregnancies, unsafe abortions, infertility, gender-based violence and sexual dysfunction [6].

In the United States, 20 million new sexually transmitted infections (STIs) occur each year, half of which are among 15–24-years-old adolescents. Sexually transmitted infections and the resulting complications can cause serious health consequences [7, 8]. Adolescent pregnancy is a global problem occurring in all countries. In Iran, the highest fertility rate has been occurred between the two age groups of 15–19 and 30–39 years old [9]. Globally, at least 10 million unintended pregnancies occur each year among 15–19-years-old adolescents in developing countries. Adolescent mothers face higher risks of complications such as eclampsia, puerperal endometritis, and systemic infections than 20–24-years-old. To get rid of the problems caused by their unwanted pregnancies, most adolescents seek abortion which is often performed unsafely. Out of the estimated 5.6 million abortions that occur each year among 15–19-years-old adolescent girls, 3.9 million are unsafe. As a result, pregnancy and childbirth complications are the leading cause of death among these girls [10].

However, there are still obstacles to the implementation of reproductive health strategies for adolescent girls in many countries, especially developing ones [11]. Under the influence of the challenges of gender inequality such as child marriage, female circumcision, incomplete high school education, lack of job security, overwork at home, less decision-making power and limited travel in the community, girls are more vulnerable to social harms than boys [12]. Moreover, inadequate information and education about gender and reproduction, insufficient access to health services, unsafe sex, less control over reproductive and sexual decisions, familiar partner violence and sexual violence make girls more vulnerable than boys to the sexually transmitted diseases [13].

In some countries such as Iran, reproductive and sexual health information and services provided by the health system are usually inappropriate for the adolescent girls, as these services are actually designed for married women. In Iran, political barriers are also among other major barriers to the provision of reproductive health for adolescent girls. Cultural and social challenges, structural and administrative barriers, and unpreparedness of the health system to provide sexual and reproductive health services to vulnerable adolescents are considered as barriers to the successful implementation of sexual and reproductive health programs for adolescents in Iran [14]. Iranian mothers’ negative attitude towards sexual health education for adolescents is another important obstacle. Lack of knowledge and communication skills are the main reasons for not talking about such issues [15]. Insufficient education and information in sexual risks, restricted and difficult access to the services, high costs of the services, lack of health insurance coverage and lack of financial independence, fear, embarrassment, inadequate knowledge, misconceptions, stigma and concern about complications and contraceptive measures, absence of a reporting system on issues such as premarital sex, induced abortion, sexual abuse or sexual coercion are among the challenges associated with the sexual and reproductive health of adolescents [16, 17]. Non-confidentiality of information during the service provision and lack of diversity in contraceptive methods are among other obstacles [18].

Iran is an Islamic and traditional country filled with strict religious rules and Iranian customs and culture. Based on the Islamic laws of Iran and other Islamic countries, premarital sex is not approved by religion, family, law and society, and out of wedlock relationship of an under 18-years-old vulnerable can often be challenging and she will be cruelly abused. Additionally, the existence of a center that provides adolescents, especially girls, with reproductive and sexual health services, is illegal and contrary to the religious rites of our country. As such, many factors affect the sexual and reproductive health of these vulnerable adolescents. Accordingly, this study aimed to explain the barriers of vulnerable adolescent girls in having access to sexual and reproductive health. To describe the sexual and reproductive health needs of vulnerable adolescent girls was other specific objective.

## Methods

### Study design

The present study was a part of an exploratory sequential mixed methods study (Qual-Quan) [19] which conducted from April 2019 to June 2020 through using content analysis approach.

### Settings, samples and recruitment

In the present study, sixteen 14–19-years-old vulnerable adolescent girls and twenty-two key informants (health providers, nurses, midwives, reproductive health professionals, obstetricians, psychologists, psychiatrists and addiction therapist), from cities of Isfahan, Tehran and Mashhad in Iran participated in the study. These girls were selected using purposive sampling method and considering the maximum variation strategy in terms of age, education, and the economic situation. A number of girls were found based on the previous experiences of the research team in identifying and referring to drug hangouts where such girls used to go; other girls were selected from the girls who were arrested by police at boy and girl joint parties or after fleeing their homes and being handed over to the 123rd Emergency Department. All of these girls were interviewed at welfare centers. In these centers, other eligible girls who had been sexually abused were also selected for interviews. After being met by us, some of these girls introduced their other friends to us and, thus, the sampling continued based on snowball sampling method. In addition, a number of midwives and nurses, health care providers, psychiatrists, gynecologists, addiction therapists and psychologists are informally interacting with the Welfare Organization regarding sexual and fertility issues as well as the psychological problems of vulnerable adolescent girls. We had access to them through the Welfare Organization. We also included reproductive health experts from university-affiliated research centers who conduct research on sexual and reproductive health and social factors affecting health. Moreover, head of the School of Health in the health center of the province was interviewed as well.

The key informants were also selected using purposive sampling method and considering the maximum variation strategy in terms of work experience and occupation. Inclusion criteria for the adolescent girls consisted of 12–19-years-old girls, Iranian citizenship, never married, onset and stabilization of menstruation and no psychological disorders; inclusion criterion for the key informants was having at least two years of work experience. After finding eligible participants, none of them refused to participate in the study. They were recruited in person or by phone calls. Tables 1 and 2 present the demographic information of the participants.

**Table 1:** Demographic characteristics of vulnerable adolescent girls

P	Age	Education level	Age of first sex	Age of first pregnancy	Gravida	abortion	Condom use	Drug use or alcohol	Receive money for sex	Parental addiction	Divorce of parents	Death of parents
1	19	diploma	13	14	2	2	–	Drug	√	father	√	–
2	17	elementary	12	14	3	2	–	Drug	–	both	√	–
3	19	High school	16	–	–	–	√	alcohol	√	father	–	–
4	19	University students	15	–	–	–	–	both	√	–	√	–
5	16	High school	13	–	–	–	–	–	–	–	–	mother
6	17	illiterate	16	17	1	1	√	–	–	–	√	–
7	18	High school	17	–	–	–	√	both	√	father	–	–
8	17/5	High school	15	–	–	–	–	both	–	–	–	father
9	15/5	High school	14	–	–	–	–	–	–	father	√	–
10	19	elementary	13	15	2	2	–	–	√	–	–	father
11	17	High school	15	–	–	–	–	alcohol	–	–	–	–
12	14	elementary	12	–	–	–	–	both	–	both	–	–
13	19	High school	11	–	–	–	–	both	√	–	–	–
14	19	High school	17	17	1	1	√	Drug	–	mother	√	–
15	19	High school	13	14	2	1	–	both	√	–	–	mother
16	18	High school	17	–	–	–	√	both	√	–	√	–

**Table 2:** Demographic characteristics of Specialists or key people involved with vulnerable adolescent girls

p	age	Education level	Field of Study	profession	gender	work experience (year)
17	35	Bachelor	Psychology	Health center consultant	woman	7
18	54	Ph.D.	Reproductive health	Academic member	woman	24
19	32	M.Sc.	Clinical Psychology	Consultant of Drop- in –Center (DIC)	woman	3
20	33	Bachelor	Psychology	Consultant of Drop- in –Center (DIC)	woman	4
21	52	Bachelor	Midwifery	Midwife (private office)	woman	27
22	42	Bachelor	Psychology	Psychologist (private office)	woman	11
23	56	specialist	Psychiatry	Academic member	Man	30
24	51	Bachelor	Midwifery	Midwife (private office)	woman	16
25	46	specialist	Obstetrician-gynecologist	Obstetrician (private office)	woman	13
26	32	M.Sc.	Clinical Psychology	Consultant (private office)	woman	5
27	46	M.Sc.	Midwifery	Midwife (private office)	woman	15
28	46	Bachelor	Health	Head of School of Health	woman	20
29	37	M.Sc.	Clinical Psychology	Consultant of Welfare Social Emergency Center	woman	5
30	37	Ph.D.	Educational Science	Psychologist of Welfare Rehabilitation Center	woman	12
31	45	Bachelor	Nursing	Nurse of Social Welfare Emergency Center	woman	12
32	35	Ph.D.	Reproductive health	Academic member	woman	14
33	40	specialist	Obstetrician-gynecologist	Obstetrician	woman	13
34	50	Ph.D.	Addiction Therapist	Head of Addiction Treatment Center	Man	20
35	37	Ph.D.	Reproductive health	Academic member (private office)	woman	12
36	56	Bachelor	Midwifery	Midwife (private office)	woman	30
37	46	Bachelor	Midwifery	Midwife (private office)	woman	20
38	46	M.Sc.	Clinical Psychology	Consultant at the Police station	Man	15

### Data collection

Data collection methods included semi-structured in-depth interviews for both adolescent girls and key informants. The third author (RP) conducted the interviews. She had 7 years of working experience in midwifery and was Ph.D. candidate in reproductive health. She had no previous contact or relationship with the participants and centers. The first and second authors had experience in qualitative studies and in the field of sexual and reproductive health. They participated in the first 10 interviews and analyzed the data. They made sure that the third author was thoroughly trained in in-depth interviews, and after making sure, the other interviews were conducted by her. All interviews were read by the first and second authors separately and it was decided to refer to the participant again in case of ambiguity, as such conditions did not occur for any of the interviews. It was also decided that the first and second authors would have 70% agreement with each other on the formation of the categories. The scheduling and location of the interviews were determined by the participants. Prior to beginning the interviews, the researcher explained the objectives of the study to the participants and obtained their written and oral consent to conduct the interview. The interviews lasted for 40 to 60 minutes, which were recorded with the permission of the participants and were immediately transcribed. The specific objectives of interviews with adolescent and key informants were: 1. To explore how unmarried adolescent girls get involved in sexual relationships, 2. To explore adolescent girls’ experiences and issues they face following such relationships, 3. To explore the experiences of key informants in dealing with and caring for vulnerable adolescent girls, 4. To explore how vulnerable adolescent girls’ sexual and reproductive health can be improved or safeguard. The interviews, then, continued with meticulous questions with regard to the provided answers. Sampling was halted when no new interview data came out.

### Data analysis

Data analysis was performed manually. After completing the first interview, the data analysis was performed using the conventional (inductive) content analysis method as explained by Graneheim et al. so the codes, subcategories and categories were generated from the data [20]. The interviews were transcribed verbatim and saved in a word document by the third author (RP). The interviews were then reviewed repeatedly by the first and second authors so that a complete understanding of them related to the research aim, can be achieved. The sentences and phrases were then inductively coded, clarifying the codes that were not clear was done by reading and going back to the original data. Similar codes were merged and merged codes with a similar meaning were grouped together to create subcategories. When no new information was obtained and the concepts extracted from the codes were repeated, we found that we had reached saturation. Thereafter, comparing the subcategories with each other, the conceptually related ones were placed in a main category. The codes, subcategories and categories were discussed in many sessions among the authors [21, 22].

### Rigor and trustworthiness

In order to evaluate the quality of the data and findings, four criteria of credibility, dependability, confirmability and transferability were used. The credibility of the data in this study increased using prolonged engagement with the data, member checking and repeated reading of the interview texts and transcriptions, peer debriefing, using complementary opinions of colleagues, writing reminders and various bracketing methods for data collection. In order to achieve data dependability, a complete and continuous method of recording decisions and activities of data collection and analysis was used, with the initial codes interpreted based on the participants’ experiences and examples of extracting categories and choosing excerpts from the transcripts of the interviews for each category. The data were also examined by an expert researcher who had no connection with the research and was an external observer. For the confirmability of the data, the entire research process and decisions were recorded by the researcher, so that others would follow the research findings if necessary. Also, the texts of some interviews, extracted codes and categories were provided to the research colleagues and a number of faculty members who were familiar with the qualitative research analysis but did not participate in the research. They were asked to examine the authenticity of the coding process and their views on categories were reviewed. Finally, for the transferability of the data, the findings were studied by several individuals who had characteristics similar to those of the participants of the study but did not take part in the present research process. These subjects were introduced by the same offices of midwives, psychologists and emergency services of welfare center (123), and were selected by the research team. With regard to the hangouts, we were helpfully introduced by the hangout manager (Mamasan) and the girls were introduced by her. During the interviews, measures including the use of face masks, physical distancing, and conducting interviews in large physical spaces were considered to prevent the spread of Covid-19. This research was approved by the Ethics Committee of Isfahan University of Medical Sciences with the ethics code of IR.MUI.RESEARCH.REC.1398.396. The participation of the participants in this study was completely voluntary and informed written consent was obtained from them. The informed consent was obtained from the parents or their legal guardian or legally appointed representatives of under 16-years-old girls.

## Results

The mean age of the adolescent girls was 17.68 years**.** The mean age of the first sexual intercourse was 14.31 years, 75% of the participating girls were addicted to drugs or alcohol, or both, and over 40% of whom had a history of parental addiction. The mean age of key informants was 43.36 years, the average work experiences was 14.90 years and most of them were woman (*n* = 19). The Demographic information of the participants are shown in Tables 1 and 2:

Findings of the present study with regard to the barriers of vulnerable adolescent girls’ access to sexual and reproductive health were explored through six main themes as follows: “family challenges”, “peer pressure”, “adolescents’ inability to make informed decisions about sex and fertility”, “lack of awareness of sexual and reproductive health threats”, “psychosocial threats to reproductive health”, and “neglecting the girls’ sexual and reproductive health” (Table 3). Identifying needs was another part of our study that the main category in our study was the need for comprehensive care of vulnerable adolescents. This comprehensive care included psychological and physical support and care and the barriers extracted to meet these needs.

**Table 3:** Barriers to Sexual and reproductive health in Vulnerable Adolescent Girls

Code	Sub-category	Main category
Undeveloped parenting style		
Troubled family	Unstable family	
Missing a parent (divorce, death, prison)		Family challenges
Family values vs. vulnerable adolescent girls	Family rejection	
Absence of effective leadership in the family		
When in Rome, do as the Romans do	Not to be left behind by the group of friends)	Peer pressure
Being obedient to the wishes of peers		
The influence of peers on beliefs, behaviors and value systems of the adolescents	Defenseless against bad peers	
Deceptive and misleading friends		
Neglect of sex training in the education system		
Absence of a sexual question and answer context in the family		
Passivity of the national media on adolescent sexual and reproductive health issues	Unanswered sexual questions	Adolescents’ inability to make informed decisions about sex and fertility
Early sexual arousal through unreliable information sources		
Inability in timely use of problem-solving skills	Defective life skills	
Weakness in correct use of communication skills with the opposite sex		
Poor awareness of using contraceptive methods		
Lack of skills in using the correct method of contraception	Unwanted pregnancy during adolescence	Lack of awareness of sexual and reproductive health threats
Lack of awareness of reproduction		
Possibility of abortions		
Lack of awareness of the risk of infertility	Lack of awareness of unsafe sex	
Lack of awareness of sexually transmitted infections		
Sexual and gender minorities	Being different from cultural norms	Psychosocial threats to reproductive health
Unrestrained sex		
Depression		
Isolation	Wounded psyche	
Rumination after sexual harassment		
Lack of organizational structure in the health system	Ineffectiveness of key organizations in providing sexual and reproductive health services	
Misbehavior of the healthcare providers		
Absence of a specialized team in welfare centers		
Unawareness of service providers at rehabilitation centers		
Lawmakers’ inattention to issues related to vulnerable adolescents		Neglecting the girls’ sexual and reproductive health
Deficiency of existing laws	Lack of legal, political and social support	
Lack of legal oversight on the health sector’s performance		
Poor performance of policymakers		
Lack of supportive policies		
Loss of golden opportunities in life caused by the lack of social support		
Lack of residential protection		
Social taboos		

### Family challenges

The adolescent girls of our study stated that unstable families and family rejection exposed them to family challenges. These challenges deprived them of a responsible family to take care of them.

#### Unstable family

Parental extremism in parenting practices, violence against adolescents, discrimination and inflexible behavior, the absence of one or both parents caused by divorce, death, or imprisonment have led to the neglect of these girls. All of these could lead to the involvement of adolescents in insecure relationships. In this regard, participant *No.*5 said:

*“I wasn’t allowed at all to go to my classmates’ homes. I couldn’t go out with them. I used to go to school from home and vice versa. Neither was I allowed to go to the nearby shops for shopping. I was under difficult conditions. I’d like to find a companion; either a girl or a boy”.*

Participant *No.*7 also said:

*“My parents are so strict. My dad bickers over everything all the time. He beats me up with everything he finds. My upper part of the lip was torn when he hit it with a curtain rod. For this, I decided to get out of home, as I was so tired”.*

In some other families, delinquent parents, economic poverty, prevalent sexual behavior, and disintegrated parental privacy against children, or rather, high-risk sexual behaviors in the family, provide the grounds for early sexual intercourse and the onset of vulnerability in the adolescents. As participant *No.*22 said:

*“Most of the time, parents themselves cause injuries, that is, they’re engaged in rampant sexual relations and extra-marital affairs. Observing such scenes, especially in a small family context, which is supposed to provide a safe haven for the kids, will cause injuries”.*

Regarding the loss or absence of one of the parents in the family, participant *No.*26 said:

*“Another group of girls had families whose parents had separated. The father had started a new life, and the mother also had a boyfriend or was remarried. Some of these girls also lost their fathers. The girls were confused, looking for a foothold, and engaged in sexual relationship with their boyfriends that even led to pregnancy and abortion.”*

#### Family rejection

The participants emphasized the role of confronting family values with them and the lack of an efficient parent, which led to ignoring the health status of adolescent girls, especially their reproductive and sexual health. These reprehensible behaviors of the family and their indifference had prevented the adolescent girls from expressing their injuries and remain silent about their endangered health. This issue, in turn, had accelerated the process of injury even more. Participant *No.* 21 said:

*“These children are so repressed in the family that even if they’ve a problem, they don’t dare to tell anyone; and when the issue is over, a 14-years-old girl with genital warts and incontinence stools goes to the midwife’s office for advice and medical services”.*

Participant *No.*13 also said:

*“I was always careful not to do vaginal sex with the boys when I was with them, because I’d to tell someone. I was both scaring and didn’t dare to talk to anyone, not even my mother. Oh, to a mother who was illiterate herself and if she knew I was having an affair with a boy, she was insulting me, what should I say, I didn’t know what to do next.”*

### Peer pressure

The adolescent girls participating in the present study stated that in their youthful longing, they needed to interact with their peers. Given the fact that their family was not adequately efficient, they needed the approval of their peers and in some cases even obeyed them.

*Not to be left behind by the group of friends*: Findings showed that being accepted in the company of friends forces the adolescent to follow the behavior of her friends. They did not want to be left unattended by their friends.

Participant *No.*16 talked about the need to be accepted by her friends:

*I remember I was in charge of the school student council and we only talked about sex and boys with my friends when we got together. I couldn’t leave them as they might reject me and I might lose my place among them. Thus, I even sometimes took part in their discussions “.*

#### Defenseless against bad peers

Being in the company of bad friends and the desire to be accepted by them has been a reason for following risky behaviors such as use of alcohol, smoking, substance abuse and unprotected sex. These deceptive and misleading friends are the agent of entering the cycle of harms. In this regard, participant *No.* 8 said:

*“I just wanted to make friendship with him, but he insisted to see some films together. Finally, after insisting and expressing some romantic words, we sat down and watched the film together; then I felt excited, something that I hadn’t experienced before. I don’t know if it was a good or bad feeling. But then we had sex”.*

Other participants referred to their being seduced by a boyfriend and having sex too. As such, participant *No.*3 said:

*“He lied to me and said it is just a simple touch. Then, during the relationship, he filmed me and threatened that he would show the film to everyone if I didn’t move the relationship forward. For 2 years now, with this movie, I’ve been caught in a relationship that I hate and every time it’s done, I hate myself.”*

Another participant referred to the surprise and defenselessness of adolescent girls in front of some of their friends:

*“They’re inexperienced and immature. They can’t protect themselves. On the other hand, being deceived by bad boys, with things like a car or romantic words or a gift, they’re trapped and because of immaturity, they’re easily and cruelly abused. Some of these adolescent girls have been sexually abused; so violently that they heal their wounds and injuries with several treatments” (participant No.36).*

A reproductive health expert maintained that these girls follow the risky behaviors of bad friends and, then, should tolerate the subsequent consequences:

*“Another point is that most girls in the company of friends tend to use drugs and use all kinds of drugs, especially stimulants. In fact, drug use can’t be separated from having sex with the opposite sex. It means that they seem to complement each other. In this situation, in order to stay in the group, one consumes drug and has unsafe sex, which may even lead to unwanted pregnancy and abortion” (Participant No. 18).*

### Adolescents’ inability to make informed decisions about sex and fertility

Unanswered sexual questions and imperfect life skills prevented the female adolescents participating in the study from making quick and accurate decisions about their sexual health and fertility.

#### Unanswered sexual questions

The participants acknowledged that they had incomplete information about sex and pregnancy, and that there was currently no suitable platform in our country to inform adolescents about reproductive health issues. Thus, their questions about sexuality had remained unanswered. In Iranian families, parents refuse to talk with their children about sexual issues or even answer their reasonable questions about sexuality and fertility in order to protect their adolescents. In this regard, participant *No. 14* said:

*“I couldn’t talk to my mother about these things. Once I talked with her about my problem but I said it was my friend’s and my mom blamed her. From then on, I talked about my boyfriends with my girlfriends whom I knew they’d also boyfriends”.*

*“Our parents think their children are going astray. They’re not taught or guided about sexuality. This causes them to be unaware of the dangers” (participant No.37).*

In the educational system, adolescents’ needs with regard to sexual issues have been ignored and the national media also overlook the sexual and reproductive health of adolescents. As such, there is no specific plan or training in this regard. Participant *No.*27 said:

“*Schools have, to some extent, entered the area of educating such issues, but it is quite limited. When talking about AIDS, they censor the issue of the disease transmission through sex and fail to warn about perils related to this disease. I think they believe this could arouse children’s desires”.*

*“These words are forbidden in school. Even our biology teacher didn’t explain much about humans until we reached the reproduction chapter. So, we’d a lot of questions, but we were embarrassed to ask. No one talked about these things either in class or in the meetings that psychologists came to us. No one had the right to speak as she might be fired” (Participant No. 15).*

Adolescents’ curiosity in this regard leads them to take refuge in the insecure context of cyberspace and social networks. Unlimited access to the virtual spaces and receiving inaccurate sexual information lays the ground for adolescents to enter the cycle of harm. Moreover, friends and peers are considered by adolescents as important sources of information that provide the ground for receiving deceptive information on sexual issues. In this regard, participant *No.* 9 stated that:

*“Only on satellite networks could you understand things very well or you’d to go to the internet to find out the answer to your questions. In TV and movies, even educators don’t talk about these issues as they believe these are taboos which may make us shameless and ruin us”.*

*I was in the six-grade when I heard my classmates talking about sexual issues. Well, that was fun. They told me how sexual relations look like, and taught me how to do it. I really wanted to experience it” (participant No.11).*

#### Defective life skills

Most vulnerable adolescent girls are unable to use communication skills properly when entering a relationship with the opposite sex and fail to manage their relationships correctly. Therefore, when faced with health-threatening situations, including request for substance and alcohol use or unprotected sex by the friends or opposite sex, they fail to manage the situation and cannot use problem-solving skills, especially the skill of saying no. This issue was explained by participant *No.*17:

*“One of them said it was in a party where girls and boys were dancing together. I hadn’t gone to such places before, but I failed to say no to my friends. Well, it was cool. I couldn’t say no. So, this can be a defective social skill”.*

*“It was as if we’d never learned to say no. For example, the first time my friend invited me to drink alcohol in presence of other boyfriends and girlfriends, I don’t know why I couldn’t resist. Because of that inability of saying on, I got involve in a lot of relationships with boys who sometimes I think were worthless and I wish I’d said no from the beginning” (Participant No. 13).*

*“I very quickly accepted any relationship and I didn’t know how to talk to the boys and which words of them I should accept and which words I shouldn’t. Thus, I entered a relationship with any boy who said he loved me and bought me something or gave me a ride” (Participant No. 10).*

These factors lead to uninformed decisions in adolescents about sex and fertility.

### Lack of awareness of sexual and reproductive health threats

Adolescents do not have awareness and a proper understanding of issues such as sexual affairs, pregnancy, contraceptive methods and the short- and long-term consequences of risky sexual behaviors such as unwanted pregnancies, recurrent miscarriages, and risk of infertility, as well as sexually transmitted infections and their complications. Owing to this lack of awareness they may act incautiously and heedlessly when they are engaged in a relationship with the opposite sex.

#### Unwanted pregnancy during adolescence

The participants stated that they did not have the necessary information about the pregnancy process and the use of contraceptive methods. Moreover, as these relationships are stigmatic, they did not have access to a center where they could receive these services. In this regard, participant *No.2* stated that:

*“I didn’t know what a sexual relation looks like. I didn’t know how I could be made pregnant when entering a relationship with the opposite sex. I’d refrain from all of this if I knew what they meant”.*

Similarly, participant *No.*1 said:

*“I didn’t know anything about contraceptive methods and I couldn’t understand how to use them. When entering a relationship, I didn’t care whether or not the other side had used condom. I also took the emergency contraceptive 10 days after sex, which was useless and I got pregnant. My mother took me to a house where they aborted my baby. I was bleeding so much that I thought I was about to die”.*

The psychologist of the Welfare Social Emergency Center said:

*“Parents should give their children a series of trainings on sexual issues, which they don’t teach because of a series of misconceptions. For example, they believe that such teachings will make their children shameless. Accordingly, their children experience sex as they’re unaware that they may be pregnant. When they become pregnant, they begin to think about a solution” (Participant No. 29).*

#### Lack of awareness of unsafe sex

Most of the adolescents participating in this study had incomplete, limited, or inaccurate information about safe sex and sexually transmitted infections. Thus, participant *No.*12 said:

*“I felt like having a sore throat, but it was gynecological infection. Because what I saw while bathing was like a sore throat when I’d a cold. I thought I must have an infection and I’d get better. Away from my mother’s eyes, I took her medicine and used it.”*

About having unsafe sex, participant *No.*7 said:

*“ I was afraid of losing my virginity before marriage. That’s why I’d anal sex with 5 of my boyfriends. I was very annoyed and I always have sores on my anus. I have also discharge. After examination, midwife told me that I’d a severe infection in my anus and that I even had some warts that I’d not noticed myself.”*

One of the participants also emphasized the need to follow up and take care of sexually transmitted infections in these adolescents and said:

*“The problem for adolescent girls with unsafe sex is that they develop sexually transmitted infections such as warts, herpes and acute proctitis. This’s followed by pain, bleeding and constipation, and eventually anal sphincter involvement. They’ll go to a gynecologist if anyone guides them” (Participant No.33).*

### Psychosocial threats to reproductive health

Psychosocial consequences following sexual orientations and unsafe sexual behaviors are other barriers that can affect the reproductive and sexual health of vulnerable adolescent girls. Furthermore, the norms of society do not accept some of these sexual orientations, and the stigma towards these adolescent girls is increasing every day. Also, unsafe sex and sexual abuse expose these girls to mental disorders.

#### Being different from cultural norms

Most adolescent girls reported they had been engaged in sexual behaviors such as multiple sex or relationship with same sex. About having multiple sex partners, participant *No.*15 said:

*“Four guys raped me. After all, I thought I’d nothing to lose and from then on, I began to have sex relations and it didn’t matter to me whether I’d fall sick or not, and then, I felt I was free to have any affair. It was as if I was punishing and hurting myself because of that stupidity”.*

With regard to same sex relationship, participant *No.12* said:

*“After having some relationships with boys, I’d a feeling that I was even willing to have a relationship with my sister to get rid of it. Ever since I came to this center (the center for caring for injured girls in the Welfare office), I’m always drawn to my roommates. I want to touch them. It’s as if when I do this, I’m a little calmer and I get rid of annoying thoughts.*

Participant *No.* 22, who was a psychologist and has repeatedly dealt with these adolescent girls, said:

*Sometimes, after having many relationships, these girls have a lot of mental problems and lose calmness of their mind. They’re constantly looking for a relationship and that’s why they’re even willing to enter a relationship with same sex. These relationships aren’t accepted in our society; this issue and taboos make them unable to use reproductive health services and counseling.*

#### Wounded psyche

Numerous psychological traumas following intercourse with the opposite sex or sexual harassment were among the barriers to the maintenance of sexual health and fertility in adolescents. Intentional or unintentional engagement in sexual intercourse has led to loss of mental and behavioral balance, rumination, and psychological problems such as depression and withdrawal in adolescent girls. Participant *No.*11 said in this regard:

*“After the second rape, I was very sad; because I’d lost my original boyfriend and I was notorious. My screams and shouts during the rape were constantly in my head. I was no longer bored with anyone. I was always with my mother and sister. I’d nightmares at night. I turned off my cell phone and wasn’t not ready to talk to anyone, I’d an anal fissure after the rape, but it didn’t matter to me to go to the doctor”.*

The psychologist spoke about the frustration of these adolescent girls:

*“First come love and the passion of youth. But because of immaturity, they can’t control their emotions. They every day enter a new relationship and constantly fail to establish these relationships. On the one hand, they feel stupid and deceived and, on the other hand, they become the subject of finger-pointing by boys and their peers. Their self-esteem is damaged by sexual conflicts, and they become indifferent to their fate and future” (participant No.26).*

### Neglecting the adolescent girls’ sexual and reproductive health

The findings showed that vulnerable adolescents have been abandoned in the high-risk context of society. The key decision-making organizations, as well as law and policymaking institutes have not provided a suitable support platform for promoting the sexual and reproductive health of vulnerable adolescents.

#### Ineffectiveness of key organizations in providing sexual and reproductive health services

Currently, key organizations, that are supposed to provide sexual and reproductive health services to vulnerable adolescents, have turned to be ineffective. The participants believed that within the national health system, there is no organizational structure to identify vulnerable adolescents and provide them with the necessary services. Participant *No* 16 said:

*“We aren’t supported anywhere. If at the health center they find out that we aren’t married and we want to be examined or even we have something in our mind and want to tell the center psychologist about our problems with our boyfriends, they don’t support us at all; they just add to our problems because they tell our parents”.*

*“We’ve no written and formulated guidelines on vulnerable adolescents. Hence, we’ve no specific plans in this regard for them”*

*(*Participant *No* 28*).*

Similarly, participant *No* 32 believed that there are no necessary policies and programs in this area:

*“We left these things to psychological health unit. The Ministry has limited us not to talk about these issues. We don’t have a specific program for these children at the ministry level”*

*.*

There are also no necessary interaction and cooperation among organizations involved in fighting social harms. Within the health system, misbehavior coupled with blame and the judgment of health service providers, on the one hand, and concerns about non-compliance and confidentiality of information, on the other, constitute the obstacles which prevent vulnerable adolescent girls from referring to the health system and talking about their problems. In this regard, participants 5 and 2 stated that:

*“I felt afraid to go to the health center for I didn’t dare to speak about my infection as they were supposed to tell my mom” (participant No.5).*

*“They thought I was addicted, and said that I smelled horribly of cigarette smoke, while I didn’t smoke at all and only took methadone. After I’d a cesarean section, anyone said in the room: ‘Are you dead? Are you alive? Do you want something? Don’t you want anything?’ I’d to do my works myself, they treated me differently. I was harassed; after 4 days, I ran away and I didn’t pursue any of the problems that happened to me after the cesarean section” (Participant No. 2).*

There is no specialized team in the field of sexual and reproductive health in welfare centers.

*“There’s only one nurse and one doctor in the welfare center where I’m. I’m a girl and ashamed to say many things to a male doctor” (Participant No. 6).*

#### Lack of legal, political and social support

Legislators and policymakers’ failure in considering the issue of sexual and reproductive health in adolescents and their improper functioning, absence of codified laws and support policies, prejudice in some existing laws, stigmatizing behavior by legal authorities in dealing with the issue, wrong judgmental attitudes on vulnerable adolescents and shortcomings in the legal monitoring of the issue by health sector in the field of sexual and reproductive health are serious barriers to the maintenance of the sexual health and fertility of vulnerable adolescents. Participant *No 35* also admitted that:

*“I know a 15-years-old girl who’d been raped several times by her father and cousins. After a while, the magistrate had voted in favor of the family as the girl, the claimant, had no witnesses. We don’t have a proper-functioning court system to pay attention to the perils facing the kids”.*

*“I’d not have had an abortion if the law had protected me from marrying a man who’d raped me and kept the fetus. I’d go to a safe place to have an abortion so that I’d not become so infected now; because if they find out that I’m pregnant, I can’t have an abortion, even if I’m pregnant by being rape. It’s not legal” (Participant No. 1).*

‌Influential organizations, NGOs and philanthropists’ failure to provide social support for vulnerable adolescents has led to the loss of golden opportunities to educate and empower the adolescents. This exacerbates the vulnerability of girls. In this regard, participants *No.*18 and 9 stated that:

*“Unfortunately, the reality is that these kids aren’t supported by any charities or benefactors because of the misconceptions that exist with regard to them. They need to be educated and supported to become a member of the society rather than become a high-profile criminal”.*

*“I couldn’t go to school anymore as I was fired because of having boyfriends. Now I also carry out the stigma of coming to welfare and no school will accept me anymore. I want to study and become a useful person, but no place accepts me” (Participant No. 9).*

Absence of legal support for these adolescents has made it impossible for them to live in a safe place until suitable living conditions are provided for them. This can expose them to traumatic context of the family and society, and their sexual and reproductive health is further compromised. Participant *No.29* also believed that:

*“But the problem is that after this short stay they’ve to go back to the family context where they don’t have a good condition. This’s because our policymaking proceedings and those who codify plans for our kids don’t support the welfare Organization at all and fail to provide safe havens for these girls so that they can stay there for a longer period of time until the family situation improves and, then, they can return to their families”.*

*“After here (welfare) I don’t know where to go as my family is in a bad situation. My father is addicted and my mother isn’t paying attention to me. Sometimes I’m even afraid to be at home with my dad, as he may sell me for drugs. I wish there was a place where I could stay as long as I could work and earn money” (Participant No. 9).*

Stigmatization of the vulnerable adolescents by the society and treating them reproachfully, social taboos, and the fear of the consequences of expressing one’s problems, lead to the adolescents’ concealment of injuries which results in their silence and indifference to their health status and exacerbates the injury.

*“The kids are afraid to express their experiences and think that nobody will accept them and they’ll not be embraced by the society. They fear of being rejected by others or their mistreatments. This’s become a big taboo for the kids.” (Participant No.20).*

*“I didn’t go to the doctor or the health center for examination, because they might find out that there’s something wrong with me and then start blaming and insulting me. I knew that if they came to know my problem, they would look at me as a bad girl, and I didn’t like this” (participant No. 4).*

## Discussion

The stable family contributes significantly to providing a safe environment for the growth, development and protection of the adolescents’ health in all areas. For a variety of reasons, however, family context can be a threat to an adolescent’s health. The strict and permissive parenting styles pave the way for the tendency towards high-risk behaviors in adolescents [23]. The authoritarian and permissive educational method had also a significant relationship with exclusion of children from the home and family space [24]. The home environment turning to an unsafe environment following the parents’ harmful behaviors can pave the way for adolescent behavioral problems such as drug abuse, moral and social deviations, educational decline or school drop-out, high-risk sex and early pregnancy. Deviant family behaviors such as addiction, prison history, high-risk sexual behaviors and so on lead to inability of overseeing children’s performance adequately, and prevent their emotional needs from being met [25]. In single-parent male adolescents, parental monitoring has been reported lower than two-parent male adolescents; affiliation with delinquent peers together with high risk behaviors has been also higher than two-parent male adolescents [26]. Moral and religious beliefs in the family can also play a key role in preventing risky behaviors and social harms in adolescents [27, 28]. Furthermore, the family rejection of vulnerable girls, parents’ inappropriate behaviors along with rebuke and humiliation, can cause adolescents to get hurt and face injuries. In Ghana, the results of a study revealed that the parents’ first reaction to the problem of pregnancy in adolescent girls is an expression of anger, exclusion of them from home or sending them to another place to prevent rumors [29].

The loss of a safe and friendly home environment helps the peer group prevail over adolescent behaviors and actions. This paves the ground for them to engage in high-risk and health-threatening behaviors. Many adolescents have stated that they obey their friends unquestionably in order to protect their friendships, be accepted by them, and escape from loneliness. They argued that they engage under the pressure and support of a peer group in high-risk behaviors such as smoking and alcohol, running away from home, or unprotected sex [30].

Another barrier to reproductive health in vulnerable adolescent girls was their inability to make informed decisions about sex and fertility. The findings showed that most families, national media, and organizations involved in adolescent health issues have not addressed adolescent’ sex education. In Islamic contexts, people’s interpretation of religious principles and the belief that sex education may stimulate premarital sex, can prevent the education of issues such as sexual and reproductive health [31]. Additionally, many mothers are opposed to extensive sex education with details to their children and the extent of maternal education to girls was found to be confined to issues related to puberty and menstruation. Of the reasons mentioned by parents in choosing this educational area, one can refer to such things as feeling ashamed, shyness and embarrassment when teaching sexual issues to their children and the fear that they may become rude and tempted to experience sexual relations [32].

After the family, using the school’s capacity can provide a good opportunity for sex education. But it seems that sex education in the public-school system across the world is a controversial topic. In Iran, the current education system faces main challenges in this regard and sex education is ignored, because premarital sexuality is not acceptable [33]. In Pakistan most of the parents and teachers believed that education of sexual affairs is in conflict with Islamic identity and ideology [34]. In contrast, in other countries, there is a strong parental support and emphasis on sex education in schools [35, 36]. From the perspective of adolescent girls, failure to receive education on sex affairs through schools leads to unprotected sex and incidence of unwanted pregnancies [37]. Under such a situation, it is natural for adolescents to resort to various media such as the Internet, satellite channels and virtual social networks to find answers to their sexual problems. Technology can facilitate easy access to sexual information sources and provide the possibility of easy search through cyberspace, easy exchange of sex content, ease of interaction and communication with the opposite sex via mobile phones and virtual networks which may arouse sex desires through online sex networks [38].

The results of the study confirm that most vulnerable adolescent girls were not able to use communication and problem-solving skills properly in their primary encounter with high-risk behaviors. The lower the life skills, the more will be the likelihood of verbal and physical aggression, criminal behavior, and alcohol and drug abuse [39].

Vulnerable adolescent girls’ unawareness of issues related to sexual and reproductive health was another obstacle in the present study. Findings of the studies in less developed countries, including Iran, on adolescents’ reproductive health suggested that a significant proportion of adolescent girls lacked sufficient knowledge on sexual and reproductive health issues, or their understanding was limited to menstruation [40]. The research among Iranian 11–14-years-old girls indicated that their level of knowledge about sexually transmitted diseases was low and AIDS was found to be the widely seen sexually transmitted disease among them [41]. Lack of knowledge of sexual and reproductive health as well as mistrust regarding contraceptive methods are the factors that influence adolescents’ decisions about not using contraceptive methods [42]. Globally, in developing countries, adolescents’ knowledge, information and education about various sexual and reproductive health issues are not enough to make well-informed decisions in this regard. This has serious consequences for the sexual and reproductive health of adolescents [16, 43].

Another important issue that is causally associated with the incidence of high-risk behaviors in vulnerable adolescence is the development of psychological disorders. Many vulnerable adolescent girls experience one or more intimate or non-intimate sexual harassment. A history of sexual harassment in adolescent girls has serious negative consequences for them. Different findings have revealed that going through an ordeal of sexual assault in adolescence is associated with mental health disorders (depression, anxiety), drug use, alcohol use and multiple sexual relations [44–46]. Sexual orientations, identities, expressions and behaviors that differ from cultural norms (sexual minoritized) may expose adolescents to adverse psychosocial and health outcomes such as victimization, depression, suicide, substance use, homelessness, sexually transmitted infections, and unplanned pregnancy. In some communities, lack of acceptance and stigmatization of specific sexual behaviors, identities, and orientations may lead to these adverse outcomes [47]. Therefore, the need for psychological support following sexual harassments, unwanted pregnancies, and abortions as well as diagnosis, treatment and follow-up of psychological disorders in vulnerable adolescents seem to be essential [48].

As all sexual and reproductive health professionals agree on the need for a comprehensive attention to adolescents’ sexual and reproductive health issues as a health priority, in the current Iranian health system, vulnerable adolescent girls are not included in health sector policies. The healthcare system lacks the necessary facilities and skilled service providers to serve and cover vulnerable adolescents. Lack of professional provider in the field of sexual and reproductive health of adolescents in the Rehabilitation Organization was also observed. The results of a systematic review show that from the perspective of service providers, clinical environment, unawareness of available services and unavailability of services are major barriers to improving the adolescents’ sexual and reproductive health. By contrast, from the perspective of adolescents, judgmental and unfriendly attitude of service providers is the key barrier to adolescents’ access and use of services [49].

Our results indicated that legal authorities have disregarded legal aspects of vulnerable adolescent girls’ sexual and reproductive health, and the existing laws have significant shortcomings in this area. In addition to the legal areas, vulnerable adolescents are also neglected in the political dimension for reasons such as severe religious, social and political pressures. In Iran government leaders and Policymakers have not accepted adolescent health as a priority due to social, cultural and religious taboos. As a result, the health sector lacks a clear policy in this regard Shariati, Babazadeh et al., [14]. Provision of good-quality and adolescent-friendly services need sound national laws and polices [50]. It seems that lack of legal and political support for adolescent sexual and reproductive health in Iran is the root cause of many problems. Therefore, our society needs to codify some policies and laws to deal with such problems.

At social level, vulnerable adolescents are not socially protected. Stigmatizing behaviors of the society, education system and healthcare providers towards vulnerable adolescents provide the conditions for more and more serious injuries. Being single as a social barrier is an obstacle for Iranian girls to access sexual and reproductive health services [51]. Iranian society’s emphasis on abstinence until marriage shapes the stigma for sexual and reproductive health issues of unmarried women and restricts their use of sexual and reproductive health services [52]. As a result, existence of weak protective laws for vulnerable adolescents, on the one hand, and the stigmatization by some NGOs, on the other, have prevented this group from taking benefits of the financial and supportive assistance provided by these organizations. Looking at the activities of non-governmental organizations supporting adolescents in other countries shows that the positive attitude of these organizations in solving problems related to social harms of adolescents helps them participate in meeting the financial and educational needs of vulnerable adolescents. Findings of a study in Tanzania demonstrated that many NGOs have informally begun to teach issues related to sexual and reproductive health as the incidence of high-risk behaviors have been increasing among 12–16-years-old adolescents. However, the negative views of some non-governmental organizations also hinder the assistance and support of vulnerable adolescents [53].

## Conclusion

Disregard for the sexual and reproductive health of vulnerable adolescents can generate irreparable consequences for society; but numerous individual, family, social, legal and political barriers in Iran have challenged the necessary platform to ensure a context to deal with this problem. Accordingly, it seems that the obstacles can possibly be overcome by developing a comprehensive and practical program and providing legal and political support for this issue.

## Data Availability

The datasets used and/or analyzed during the current study available from the corresponding author on reasonable request.
